# Iron Acquisition Strategies of *Vibrio anguillarum*

**DOI:** 10.3389/fcimb.2017.00342

**Published:** 2017-07-25

**Authors:** Yingjie Li, Qingjun Ma

**Affiliations:** ^1^Key Laboratory of Experimental Marine Biology, Institute of Oceanology, Chinese Academy of Sciences Qingdao, China; ^2^Laboratory for Marine Biology and Biotechnology, Qingdao National Laboratory for Marine Science and Technology Qingdao, China

**Keywords:** *Vibrio anguillarum*, siderophore biosynthesis, siderophore secretion, iron uptake, iron release, iron acquisition mechanism

## Abstract

The hemorrhagic septicemic disease vibriosis caused by *Vibrio anguillarum* shows noticeable similarities to invasive septicemia in humans, and in this case, the *V. anguillarum*–host system has the potential to serve as a model for understanding native eukaryotic host–pathogen interactions. Iron acquisition, as a fierce battle occurring between pathogenic *V. anguillarum* and the fish host, is a pivotal step for virulence. In this article, advances in defining the roles of iron uptake pathways in growth and virulence of *V. anguillarum* have been summarized, divided into five aspects, including siderophore biosynthesis and secretion, iron uptake, iron release, and regulation of iron uptake. Understanding the molecular mechanisms of iron acquisition will have important implications for the pathogenicity of this organism.

## Introduction

The Gram-negative bacterium *Vibrio anguillarum* is a pathogen that causes vibriosis with lethal hemorrhagic septicemia in aquatic animals worldwide (Toranzo et al., 2005). Although up to 23 O serotypes of *V. anguillarum* are identified in the European serotyping system, with most serotypes encompassing free-living environmental strains (Pedersen et al., 1999), only serotypes O1, O2, and partial O3 are found to be implicated in vibriosis outbreaks (Toranzo et al., 2005). Many studies have been performed in an attempt to understand the virulence mechanism in *V. anguillarum*. Several main virulence factors have been recognized by using genetic approaches, including iron acquisition components (Naka and Crosa, 2011), hemolysins (Hirono et al., 1996; Rodkhum et al., 2005; Rock and Nelson, 2006; Li et al., 2008; Xu et al., 2011; Mou et al., 2013), metalloproteases (Milton et al., 1992; Yang et al., 2007; Varina et al., 2008; Mo et al., 2010), chemotaxis and motility (O'Toole et al., 1999; Ormonde et al., 2000), exopolysaccharides (Croxatto et al., 2007), and lipopolysaccharides (Welch and Crosa, 2005). Among them, iron uptake systems are a critical component for infection of the host fish leading to disease (Wolf and Crosa, 1986).

*V. anguillarum*, like most other organisms, has an absolute requirement for iron to synthesize a large number of crucial enzymes, which are involved in many fundamental cellular processes, such as cytochromes for cell respiration, ribonucleotide reductase for the biosynthesis of DNA precursors, and enzymes for the tricarboxylic acid (TCA) cycle (Crosa et al., 2004). However, due to the low solubility of iron (~10^−18^ M) at physiological pH in aerobic environments, ferric iron mainly forms insoluble hydroxides, whereas a cytoplasmic iron concentration of ~10^−6^ M is required for bacterial growth (Hantke, 1981). Therefore, iron is suggested to be the growth-limiting factor in ocean environments (Martin et al., 1991). To respond to this selective pressure, bacteria have evolved numerous mechanisms for iron acquisition, including transport of iron from the mammalian iron carriers, transferrin and heme, and synthesis of small ferric iron-binding molecules, known as siderophores. Some of these iron transport systems are conserved among all *Vibrio* species, reflecting their common ancestry, while other acquisition systems appear to have been developed by horizontal transfer, such as the anguibactin transport system that is mainly specific to *V. anguillarum*. It has been shown that *V. anguillarum* harbors a number of genes encoding for iron uptake and regulation, which are essential for its virulence beyond simple iron chelation (Lemos and Osorio, 2007; Naka et al., 2013b). In this article, we describe the developments in understanding the molecular mechanisms of iron acquisition systems in *V. anguillarum*, divided into the following aspects: siderophore biosynthesis and secretion, iron uptake, iron release, and regulation of iron uptake.

## Siderophore biosynthesis

Two different siderophore-dependent systems have been identified in *V. anguillarum* strains. One is mediated by a 65 kb pJM1 plasmid, which contains most of the genes encoding for biosynthesis and transport proteins of the siderophore anguibactin (Naka et al., 2013b). This anguibactin system is only found in pathogenic plasmid-bearing strains of serotype O1. The other system, existing in all serotype O2 strains tested thus far, and some plasmidless serotype O1 strains, synthesizes a catecholated-type siderophore, vanchrobactin (Alice et al., 2005; Balado et al., 2006).

### Biosynthesis of anguibactin

**Figure 1:**
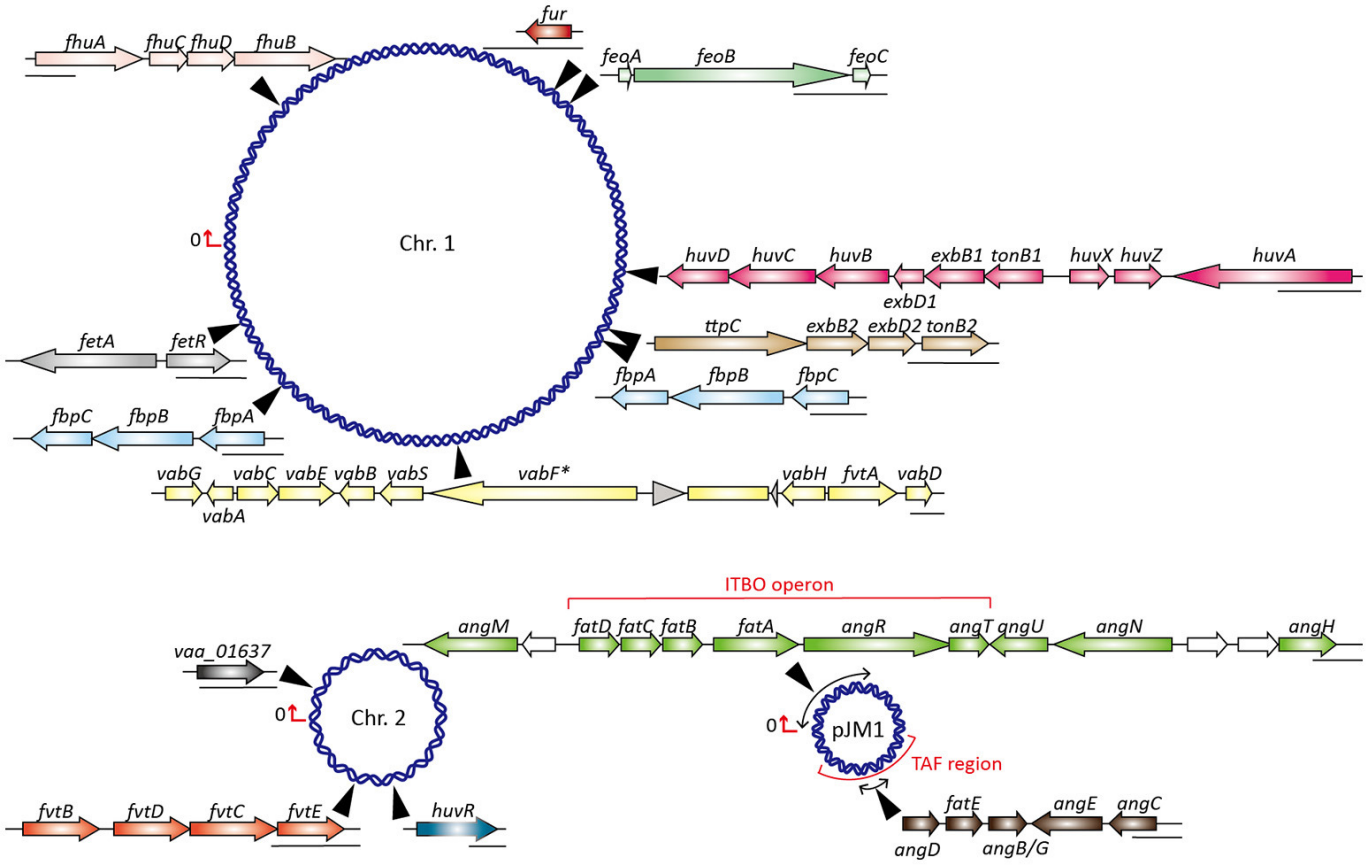
Map of *V. anguillarum* 775 chromosomes and plasmid pJM1 showing locations and molecular organization of known and putative iron transport genes. Chr., chromosome; ITBO, iron transport biosynthesis operon; TAF, *trans-*acting factor; vabF^*^ indicates *vabF* is inactivated by the insertion sequence RS1.

**Figure 2:**
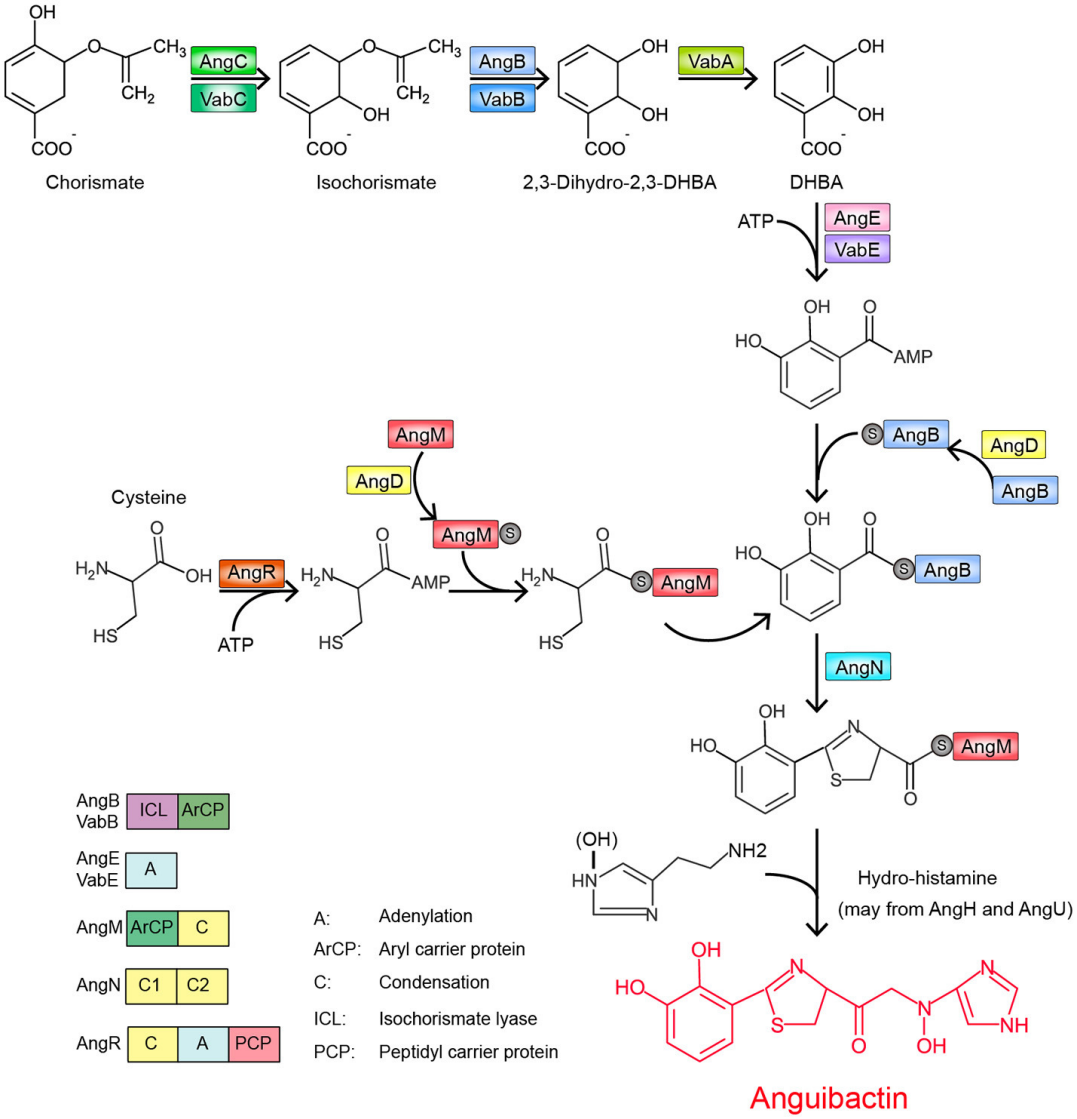
Proposed pathway of anguibactin biosynthesis in *V. anguillarum* plasmid-carrying strains (modified from Naka et al., 2013b).

The structure of anguibactin is unique in containing both catechol and hydroxamate metal-chelating functional groups (Actis et al., 1986), derived from 2,3-dihydroxybenzoic acid (DHBA) and *N*-hydro-histamine, respectively. Utilizing the chorismate, plasmid-carrying *V. anguillarum* strain 775 generates a repertoire of molecules through a ribosome-independent process and finally synthesizes anguibactin. This biosynthesis is controlled by a number of genetic determinants. To date, more than 10 different genes have been described and yield anguibactin-related phenotypes in plasmid-carrying *V. anguillarum* strains when mutated by genetic approaches (Table S1). Most are located on the plasmid pJM1 or pJM1-like plasmids while some are on the chromosomes (Figure 1). First, chorismate is catalyzed stepwise by a series of proteins, AngC/VabC (isochrorismate synthase), AngB/VabB (isochrorismatase; Du et al., 2017), and VabA (2,3-dihydro-2,3-dehydroxybenzoate dehydrogenase), to synthesize DHBA (Figure 2; Alice et al., 2005; Balado et al., 2008). In later steps, phosphopantetheine transferase AngD is required to transfer a phosphopantetheinyl moiety to a serine residue of AngB and AngM (Balado et al., 2008). AngE/VabE (2,3-dehydroxybenzoate-AMP ligase) activates DHBA to form acyl adenylate and further transfers it to the free thiol of the phosphopantetheine AngB (Liu et al., 2004; Alice et al., 2005). AngC, AngB, and AngE are pJM1-encoded proteins while VabC, VabB, VabA, and VabE are chromosomally encoded with VabC, VabB, and VabE showing functional redundancy with AngC, AngB, and AngE, respectively. AngN catalyzes this DHBA thioester to combine with cysteine, which is activated by AngR and tethered by AngM, thereby producing a dihydroxyphenyl-thiazoline-thioester. AngH (histidine decarboxylase; Tolmasky et al., 1995; Barancin et al., 1998) and possibly AngU (Naka et al., 2013b) modify the histidine to form *N*-hydro-histamine, which is then transferred to dihydroxyphenyl-thiazoline-thioester to yield anguibactin. It is interesting to note that homologs of these plasmid-located genes are all present on the chromosome of *Vibrio harveyi*, which is also able to produce anguibactin, suggesting that the plasmid-mediated anguibactin system might originate from *V. harveyi* or vice versa (Naka et al., 2013a,b).

### Biosynthesis of vanchrobactin

**Figure 3:**
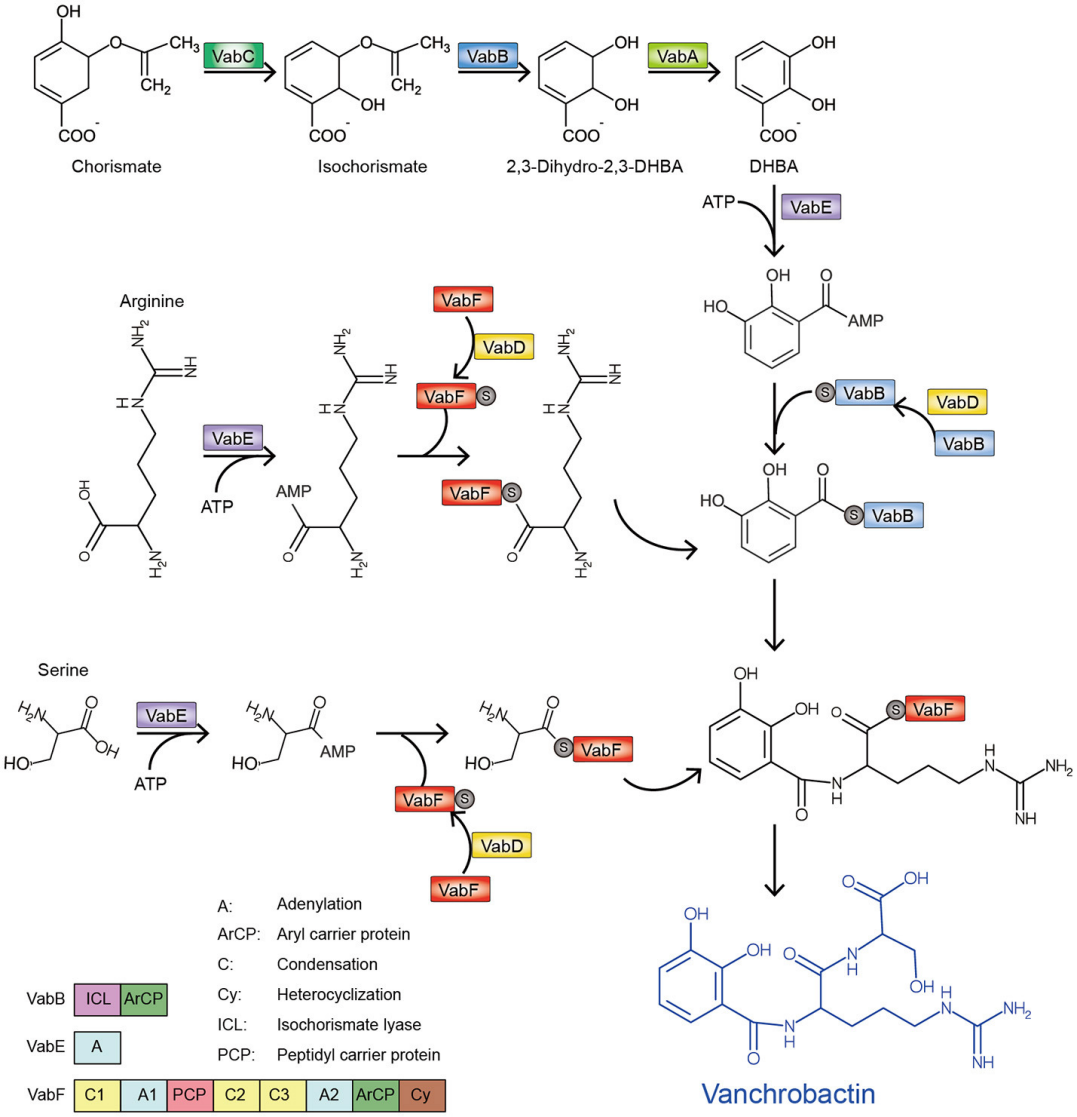
Proposed pathway of vanchrobactin biosynthesis in *V. anguillarum* plasmidless strains (modified from Balado et al., 2006).

Plasmidless O1 strains and those belonging to a number of other *V. anguillarum* serotypes synthesize a chromosome-mediated siderophore, vanchrobactin (Lemos et al., 1988; Soengas et al., 2008). In a similar way to anguibactin synthesis, DHBA of these *V. anguillarum* strains is also produced from chorismate by the sequential activities of VabC, VabB, and VabA. However, how vanchrobactin is synthesized from the DHBA precursor remains obscure, even though some genes have been found to be indispensable for this process, including *vabB, vabD, vabE*, and *vabF* (Balado et al., 2006, 2008). According to well-studied pathways for synthesis of anguibactin and vibriobactin, a siderophore produced by *Vibrio cholerae*, late steps for vanchrobactin formation in *V. anguillarum* have been proposed during which DHBA is assembled (Figure 3; Balado et al., 2006). Specifically, VabD contributes to transfer of a phosphopantetheinyl moiety to the aryl carrier domain of VabB and the peptidyl carrier domain of VabF, respectively. Like anguibactin formation, VabE activates DHBA and arginine to yield acyl adenylates, and then delivers them to VabB and VabF, respectively. The DHBA-VabB is combined with arginine by the condensation domain of VabF to form (2,3-dihydroxybenzoyl)argininate. Finally, the condensation domain of VabF in (2,3-dihydroxybenzoyl)argininate-VabF may be loaded with VabE-activated serine, which adheres to the peptidyl carrier domain of VabF to produce vanchrobactin. Although similar roles of AngB and VabB are proposed in these processes, the aryl carrier protein (ArCP) domain of VabB is not able to complement the function of the AngB ArCP domain (Di Lorenzo et al., 2011), suggesting different but unknown roles may occur between AngB and VabB during anguibactin and vanchrobactin formation.

## Siderophore export

Siderophore secretion is an essential step in iron uptake, yet the mechanisms of this process remain largely unknown. Two siderophore export systems have been found so far, including the ATP-dependent efflux pump and the major facilitator superfamily protein (MFS)-mediated efflux pump. PvdRT-OpmQ is the first ATP-dependent export system to be identified in *Pseudomonas aeruginosa* (reviewed by Schalk and Guillon, 2013). Schalk and colleagues found that this system exports not only the newly synthesized mature siderophore pyoverdine but also pyoverdine that has already delivered iron into the bacterium (Hannauer et al., 2010; Yeterian et al., 2010). In addition, PvdRT-OpmQ can secrete unwanted metal-pyoverdine complexes into the periplasm of *P. aeruginosa* (Hannauer et al., 2012). By using the respective protein sequences from *P. aeruginosa* as a query in BLASTP analysis, only genes encoding for PvdR and PvdT are identified (Table S2) while the *ompQ* gene is absent in the genomes of the sequenced *V. anguillarum* strains, suggesting that the MFS system rather than PvdRT-OpmQ may participate in siderophore secretion in *V. anguillarum*.

**Figure 4:**
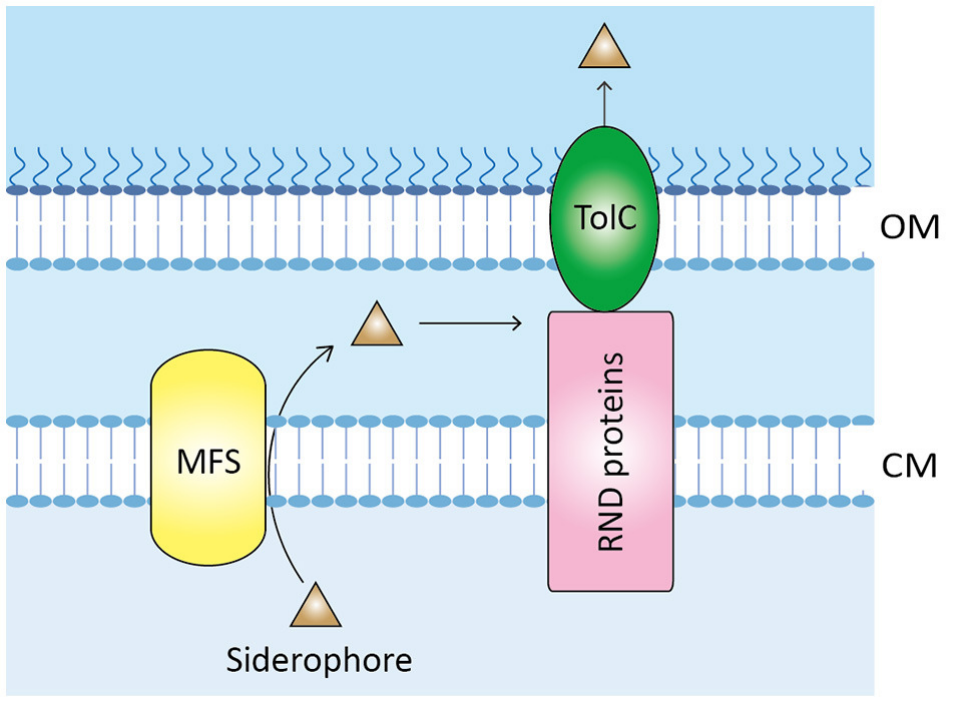
Proposed model of siderophore export in *V. anguillarum*. When produced in the cytoplasm, siderophores such as anguibactin or vanchrobactin are exported to the periplasmic space by MFS protein. Subsequently, RND proteins capture and further secrete the siderophores to the environment through the outer membrane protein TolC. OM, outer membrane; CM, cytoplasmic membrane; MFS, major facilitator superfamily protein; RND, resistance-nodulation-cell division.

The secretion of enterobactin in *Escherichia coli* is the best-studied paradigm of siderophore export via the MFS system (Horiyama and Nishino, 2014). Based on this, a proposed siderophore-export pathway is depicted in Figure 4, and putative genes involved in this process are listed in Table S2. First, siderophores are exported to the periplasm from the cytoplasm via the MSF VabS, a homolog of *E. coli* EntS that has been shown to transport enterobactin across the cytoplasmic membrane (Furrer et al., 2002). Subsequently, the resistance-nodulation-cell division (RND) family proteins, which have been found to play a role in multidrug resistance in many microbes including *V. cholerae* strains (Rahman et al., 2007; Bina et al., 2008), capture the periplasmic siderophores and secrete them to the environment via the outer membrane channel TolC. In agreement with this model, a RND efflux system recently described in *V. cholerae* plays an essential role in maintenance of cellular homeostasis by secreting the siderophore vibriobactin (Kunkle et al., 2017), which again indicates that a MFS-mediated efflux pump system might be used for siderophore export in *V. anguillarum*.

## Iron uptake

**Figure 5:**
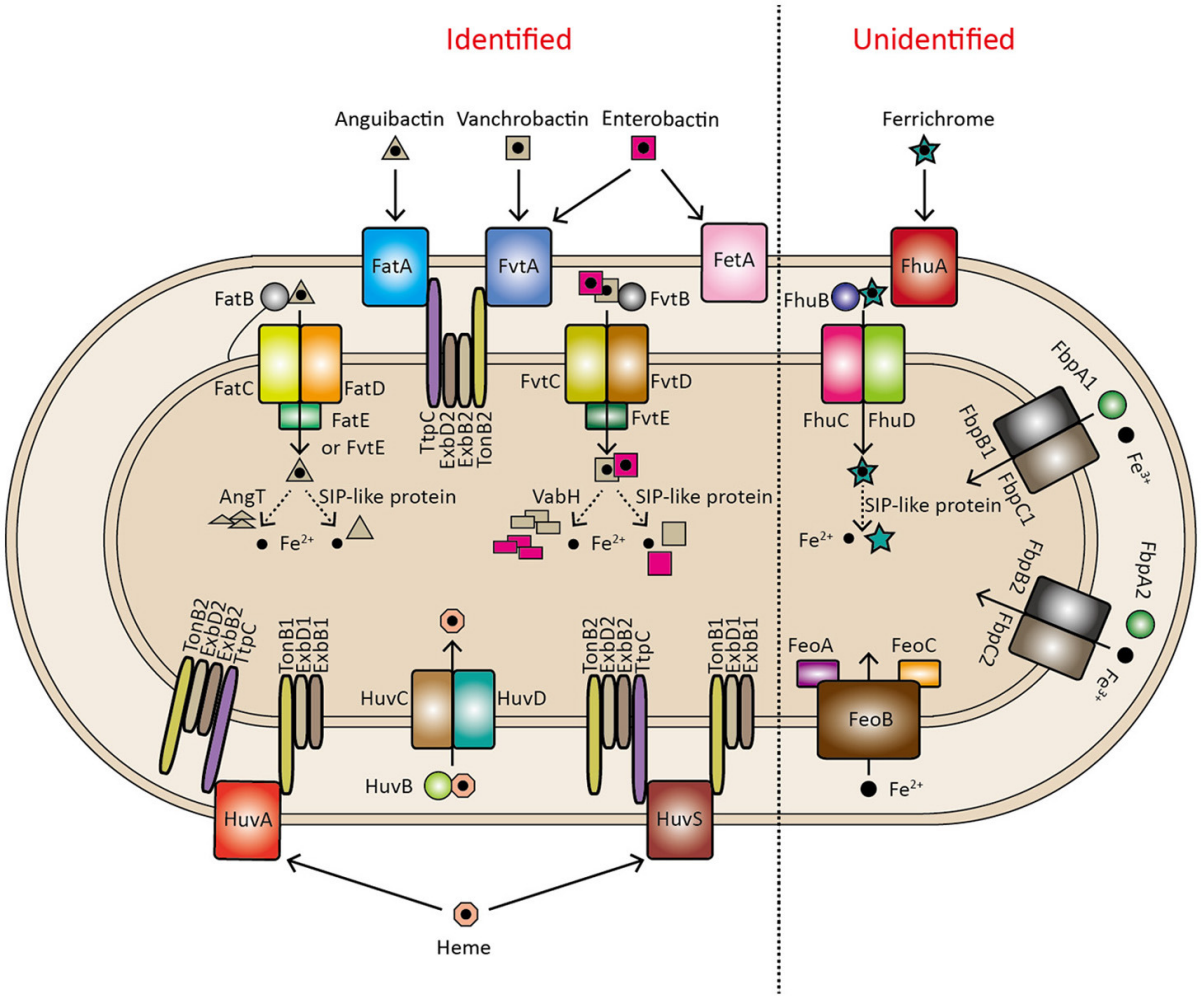
Identified and unidentified iron transport systems in *V. anguillarum*. A ferrisiderophore release model is proposed, indicated by a dashed line, including two mechanisms: degradation of the siderophore (achieved by AngT for anguibactin hydrolysis and VabH for vanchrobactin hydrolysis) and reduction of the ferrisiderophore [achieved by a siderophore-interacting protein (SIP)-like protein].

*V. anguillarum* strains contain several iron transport systems to sequester the different sources of iron, including anguibactin or vanchrobactin, heme, free Fe^2+^, free Fe^3+^, and ferrichrome, which are summarized in Figure 5.

### Ferrisiderophore import

Once siderophores are produced and exported to the environment, they capture iron to form a ferric-siderophore complex, which is recognized by a specific transporter on the surface of the outer membrane. In *V. anguillarum*, ferric-anguibactin is translocated across the outer membrane via its specific transporter FatA, which is essential for ferric-anguibactin uptake (Walter et al., 1983; Actis et al., 1988; Lopez and Crosa, 2007; Lopez et al., 2007). This process requires the TonB2 system for energy transmission, which originates from the proton-motive force of the inner membrane (Stork et al., 2004).

The TonB2 complex is located across the inner membrane and comprises TonB2, ExbB2, ExbD2, and TtpC (TonB2 complex-associated transport protein C), all of which are indispensable for ferric-anguibactin import to the periplasmic space. Deletion of either of these genes completely abolishes ferric-anguibactin uptake, and they are thereby considered essential virulence factors for *V. anguillarum* since ferrisiderophore transport during iron uptake is a critical step for virulence (Stork et al., 2004, 2007). Notably, TtpC which shows homology to the TolR protein of *E. coli*, has been identified to be part of the TonB2 system in several *Vibrio* species, including *V. anguillarum* (Stork et al., 2007), *V. cholerae* (Stork et al., 2007), *Vibrio alginolyticus* (Wang et al., 2008), *Vibrio parahaemolyticus* (Kuehl and Crosa, 2010), and *Vibrio vulnificus* (Kuehl and Crosa, 2009). Despite a high similarity among all pathogenic vibrios studied thus far, the TtpC proteins likely play an important role in specific iron transport mediated by the TonB2 system, a conclusion drawn based on the observation that *V. anguillarum* Δ*ttpC* cannot be complemented by the TtpC protein from *V. cholerae* (Stork et al., 2007; Kuehl and Crosa, 2009).

In addition to anguibactin transport, the TonB2 system is also involved in the uptake of vanchrobactin and the xenosiderophore (siderophore produced by other organisms) enterobactin. The transport of vanchrobactin and enterobactin is mediated by another outer membrane transporter, FvtA, which displays vanchrobactin-dependent expression (Balado et al., 2008, 2009; Naka et al., 2008). In the serotype O2 *V. anguillarum* strain RV22, loss of *fvtA* leads to impaired growth under iron-limiting conditions due to incapability of vanchrobactin transport in cells (Balado et al., 2009). Besides FvtA, during transport of the xenosiderophore enterobactin, it seems another transporter, FetA, is present that shows specific binding to enterobactin, and the expression of FetA is regulated by FetR (Naka and Crosa, 2012). This *fetA-fetR* cluster in *V. anguillarum* is speculated to derive from *V. cholerae* based on similar molecular organization and high similarity (Naka and Crosa, 2012).

Subsequently, when combined with the periplasmic lipoprotein FatB, ferric-anguibactin passes through the cytoplasmic membrane by using an ATP-binding cassette (ABC) transporter, which includes inner membrane permeases consisting of a heterodimer of FatC and FatD, and an ATPase FatE (Köster et al., 1991; Actis et al., 1995; Naka et al., 2010, 2013c). FatBCD are required for ferric-anguibactin transport while a *fatE* mutant is still capable of ferric-anguibactin transport due to the presence of its homolog FvtE (Naka et al., 2013c). Double deletion of *fatE* and *fvtE* significantly impairs ferric-anguibactin uptake (Naka et al., 2013c). Similarly, the passage of vanchrobactin through the inner membrane is also achieved in a stepwise manner by a periplasmic protein, FvtB, and an ABC transporter, FvtCDE, which together are essential for ferric-vanchrobactin or ferric-enterobactin import; deletion of either protein causes a defect in ferric-vanchrobactin and ferric-enterobactin transport (Naka et al., 2013c). Different from the specific involvement of FatE in the uptake of anguibactin, FvtE can transport both anguibactin and vanchrobactin/enterobactin (Naka et al., 2013c).

It is noteworthy that although *fatDCBA* encoding for ferric-anguibactin transport are located on the pJM1-type plasmid in serotype O1 strains, they are also present on the chromosomes of plasmidless vanchrobactin-synthesizing strains, albeit *fatA* is inactivated by an insertion sequence (Bay et al., 2007; Balado et al., 2009). In fact, gene clusters coding for vanchrobactin synthesis and uptake are ubiquitous in both anguibactin- and vanchrobactin-producing *V. anguillarum* strains (Balado et al., 2009), while plasmid-harboring strains do not produce vanchrobactin due to an inactivated chromosomal *vabF* gene (Figure 1; Naka et al., 2008). This is caused by the presence of the insertion sequence RS1, observed in all serotype O1 strains carrying a pJM1-type plasmid, and when RS1 is removed, pJM1-containing strains can simultaneously produce the siderophore vanchrobactin (Naka et al., 2008). However, because anguibactin appears to have a higher affinity for iron than does vanchrobactin, it is suggested to be the primary siderophore for iron import even though vanchrobactin is produced in the cell (Naka et al., 2008). Nevertheless, the minimal inhibitory concentration (MIC) values for iron chelators in plasmid-bearing strains seem to be lower than those of plasmidless *V. anguillarum* strains, which might be caused by higher stoichiometry of vanchrobactin in the plasmidless strains (Conchas et al., 1991).

### Heme uptake

Heme usually exists abundantly but not freely as a chemical compound in host cells. Therefore, bacteria secrete exotoxins such as cytolysins, proteases, or hemolysins to release it for further uptake. In *V. anguillarum*, several hemolysin genes have been identified and some of them are involved in *V. anguillarum* virulence (Hirono et al., 1996; Rodkhum et al., 2005; Rock and Nelson, 2006; Li et al., 2008, 2013; Xu et al., 2011). After its release, free heme is captured by a putative outer membrane transporter, HuvA, which has been shown to be essential for heme uptake because the *huvA* mutant does not grow when heme is used as the sole iron source (Mazoy et al., 2003). However, compared with the wild type, the *huvA* mutant still maintains heme-binding activity under the conditions tested (Mazoy et al., 2003), indicating that HuvA is not the only protein capable of binding heme. In line with this, two heme-binding proteins with molecular masses of 39 and 37 kDa were isolated from *V. anguillarum* serotype O1 and O2 strains, respectively, which are completely different from the 79 kDa HuvA protein (Mazoy and Lemos, 1996b; Mazoy et al., 1996). Therefore, Mazoy et al. speculate that, besides HuvA, additional proteins are probably present in *V. anguillarum* and function in heme binding, but not in its transport (Mazoy et al., 2003; Lemos and Osorio, 2007). Like siderophore import, this process is also energy-dependent, where HuvA is energized by TonB systems. The difference is that not only is the TonB2 complex involved in heme utilization, but a TonB1 system composed of TonB1-ExbB1-ExbD1 is also available for energy supply (Stork et al., 2004). Deletion of all genes for these two TonB systems leads to a complete defect in heme uptake and thus avirulence to the host, suggesting a key role of TonB systems in *V. anguillarum* virulence (Stork et al., 2004). The recent structural studies of the Ton complex from *E. coli* provide a mechanistic insight into this complex (Celia et al., 2016). It is proposed that the functional unit of the Ton complex contains an ExbB pentamer, an ExbD dimer, and at least one TonB. Electrophysiology experiments suggest that the ExbB-ExbD forms pH-sensitive channels, by which the Ton complex likely harnesses the proton-motive force for energy production and transduction.

Bioinformatic studies indicate that genes of the two TonB systems exist ubiquitously among all *Vibrio* species, and moreover, a third TonB system is even observed in some vibrios and other marine organisms (Kuehl and Crosa, 2010; Kustusch et al., 2011). However, it remains unclear why so many TonB systems are present in the *Vibrio* species. The *V. anguillarum* strains lack HuvA but contain an alternative heme transporter, HuvS, which is able to restore heme transport in a *huvA* mutant (Mouriño et al., 2005). The observation that *huvS* and *huvA* possess similar flanking DNA sequences implies that horizontal transmission and recombination might have occurred and thus be responsible for this genetic diversity (Mouriño et al., 2005).

Furthermore, the *huvA* gene is located in a gene cluster coding for nine heme uptake-related proteins, including HuvA, HuvZ, HuvX, TonB1, ExbB1, ExbD1, HuvB, HuvC, and HuvD (Mouriño et al., 2004). The periplasmic heme-binding protein HuvB delivers periplasmic heme to an inner membrane complex consisting of a permease, HuvC, and an ATPase, HuvD, which subsequently transport heme into the cytosol (Mouriño et al., 2004). Therefore, HuvBCD are required for heme transport, and deletion of either gene results in heme transport deficiencies (Mouriño et al., 2004). HuvZ also plays an import role in heme uptake, and loss of *huvZ* severely affects the growth of cells when heme serves as the sole iron source (Mouriño et al., 2004). Little is known about the function of HuvZ in heme utilization, and in *V. cholerae* it is suggested to have a role in heme storage (Wyckoff et al., 2004). HuvX, a predicted intracellular heme delivery protein in *V. cholerae* (Sekine et al., 2016), is not required for heme uptake in the *V. anguillarum* 775 plasmidless avirulent strain because deletion of *huvX* does not cause obvious differences in growth and heme utilization compared with the wild type (Mouriño et al., 2004). However, it is still under debate whether heme utilization is indeed involved in *V. anguillarum* virulence in nature.

### Other iron acquisition systems

Besides the anguibactin/vanchrobactin and heme uptake systems, four operons encoding putative iron transport systems, including transport of unchelated ferrous (*feoABC*) and ferric iron (*fbpABC1* and *fbpABC2*), and siderophore ferrichrome transport (*fhuABCD*), have been identified in the genomes of *V. anguillarum* strains (Figures 1, 5). The presence of different iron transport systems in *V. anguillarum* probably results from differences in growth conditions because siderophores can only promote growth under limited iron conditions that must be insufficient for iron uptake under all environmental conditions. In line with this, all the vibrios examined have been shown to have additional iron acquisition systems (Table S3; Payne et al., 2016).

The ferrous iron transporter FeoABC is speculated to be the most ancient iron transport system and widely found among bacterial species including *Vibrio* species. In *V. cholerae*, all *feoABC* genes are required for ferrous iron uptake although their functions have not been fully characterized (Wyckoff et al., 2006; Weaver et al., 2013). However, it is still unknown how ferrous iron passes through the outer membrane for transport by the Feo system in the periplasm. FbpABC, a ferric iron transporter, is also found in vibrios and has been shown to promote better growth at alkaline pH in *V. cholerae* (Peng et al., 2016). FhuABCD, responsible for siderophore ferrichrome utilization, have been demonstrated to be required for ferrichrome utilization in *V. parahaemolyticus* and *V. cholerae* (Rogers et al., 2000; Funahashi et al., 2009).

Notably, although it has been demonstrated that *V. anguillarum* is capable of using ferric citrate as the only iron source by a siderophore-independent mechanism (Mazoy et al., 1997), we do not find any genes involved in ferric citrate transport in the genome of *V. anguillarum* or other vibrios. In *P. aeruginosa*, iron delivered by citrate is suggested to enter the cell as Fe^2+^, and FeoB is further required for citrate-mediated Fe^2+^ uptake (Marshall et al., 2009). Therefore, it is plausible that FeoB of *V. anguillarum* may also play a role in Fe^2+^ uptake from ferric citrate to maintain iron homeostasis.

## Iron release from ferrisiderophores

When iron–siderophore complexes are transported into the cell cytoplasm, bacteria can use different strategies to release iron:

– Via the reduction of ferric iron.
– Via the degradation or modification of siderophores.
– Via both iron reduction and siderophore degradation.

Genes predicted for iron reduction and siderophore degradation occur in the genome of *V. anguillarum*, and proposed pathways are shown in Figure 5. VabH, a putative cytoplasmic esterase, exhibits homology to *E. coli* Fes, which can hydrolyze enterobactin during iron release (Brickman and McIntosh, 1992). Therefore, VabH may serve as a vanchrobactin degradation enzyme during ferric-vanchrobactin compound dissociation. Similarly, a putative thioesterase gene, *angT*, may be involved in iron release from ferric-anguibactin. However, deletion of *vabH* or *angT* does not completely block siderophore uptake (Wertheimer et al., 1999; Balado et al., 2006), indicating that additional pathways for iron release may occur. In accordance with this, a nicotinamide adenine dinucleotide phosphate (NADPH)-dependent ferric reductase has been identified in the genomes of *V. anguillarum* strains. It shows high homology to the reported siderophore-interacting proteins (SIP) YqjH of *E. coli* and FscN of *Thermobifida fusca*, which are suggested to participate in iron reduction during ferrisiderophore dissociation (Miethke et al., 2011; Li et al., 2015). Furthermore, Mazoy and Lemos observed that ferric reductase activities of cell fractions are significantly increased in the presence of NADPH compared to its absence (Mazoy and Lemos, 1996a), indicating a functional NADPH-dependent ferric reductase may occur in *V. anguillarum*. In addition, they also found it is only in the cytoplasmic, but not in periplasmic or membrane faction, where ferric reductase activity is stimulated under iron-limiting conditions (Mazoy and Lemos, 1996a). This implies iron release from ferrisiderophore might happen in the cytoplasm. Further investigation of the role of SIP in the ferrisiderophore dissociation pathway will provide more detail to elucidate the strategies deployed by *V. anguillarum* to release iron for utilization.

## Regulation of iron transport

As excess iron is lethal and may lead to oxidative damage to DNA when free Fe^2+^ reacts with hydrogen peroxide via the Fenton reaction (Imlay, 2002), tight regulation of iron transport is a prerequisite to meet, but not exceed, the requirement for iron. To date, several regulators have been identified in *V. anguillarum* to control the uptake of the iron, including the negative regulators Fur and an antisense RNA (RNAα), and the positive regulators AngR, TAFr, and anguibactin.

In Gram-negative bacteria, Fur is the major global regulator of iron metabolism, which serves as a sensor of intracellular iron concentration. It can bind to a Fe^2+^-bound dimer at a specific site, termed the Fur box, in the promoter region and thereby negatively regulate the transcription of corresponding genes (Troxell and Hassan, 2013). Therefore, it is not surprising that the Fur protein of *V. anguillarum* blocks or depresses the expression of most genes involved in iron acquisition systems, such as those coding for anguibactin and vanchrobactin synthesis (Salinas and Crosa, 1995; Chen et al., 1996; Di Lorenzo et al., 2004; Alice et al., 2005; Balado et al., 2008), TonB systems (Mouriño et al., 2006), and iron transport systems (Waldbeser et al., 1993; Tolmasky et al., 1994; Chen and Crosa, 1996; Chai et al., 1998; Mouriño et al., 2006; Balado et al., 2008; Naka and Crosa, 2012). In addition to Fur, an antisense RNA, RNAα, is capable of negatively modulating the expression of the *fatA* and *fatB* genes involved in iron transport by specifically binding to *fatA* and *fatB* mRNAs and thus repressing their transcription under iron-rich conditions (Waldbeser et al., 1995; Chen and Crosa, 1996). Moreover, the Fur protein is also crucial for RNAα synthesis and regulates its transcription initiation, which is independent of the iron status of the cell (Chen and Crosa, 1996).

AngR is a bifunctional protein, involved not only in the formation of anguibactin but also in the positive regulation of transport and biosynthesis genes (Salinas et al., 1989; Singer et al., 1991). Although AngR consists of two helix-turn-helix (HTH) regulatory motifs, only the first HTH is essential for gene regulation, as demonstrated by the finding that modulation of an *angR* deletion mutant is restored by a construct containing a frame shift and leaving only the first HTH motif (Wertheimer et al., 1999). These data again suggest that AngR plays an important role in both anguibactin synthesis and regulation of gene expression. The transcription of genes including the iron transport biosynthesis operon (ITBO; Figure 1) and *angN* is tightly controlled by AngR, and they display the highest expression level when iron is limited (Actis et al., 1995; Chen and Crosa, 1996; Di Lorenzo et al., 2008). The TAF (*trans-*acting factor) region shown in Figure 1 is essential for anguibactin biosynthesis and for maximal expression of the ITBO genes, which are achieved by two separate entities: one involved in anguibactin biosynthesis (TAFb) and the other in regulation (TAFr) (Tolmasky et al., 1988; Welch et al., 2000). Studies have demonstrated that TAFr and AngR work in a synergistic manner to modulate the level of anguibactin synthesis under iron-limiting conditions (Salinas et al., 1989; Salinas and Crosa, 1995). In addition, anguibactin by itself is also capable of increasing the transcriptional level of the ITBO, which reaches the highest level when AngR, TAFr, and anguibactin are all acting synergistically (Chen and Crosa, 1996).

Besides iron, many other environmental factors have been identified in vibrios, including *V. anguillarum*, that affect the expression of genes associated with iron transport, such as oxygen, temperature, carbon sources, and quorum sensing molecules (Mou et al., 2013; Payne et al., 2016). Therefore, an expanded search for sensors involved in other environmental signals may lead to a more complete picture of the strategies of iron regulation in *V. anguillarum*.

## Outlook

Despite the challenges of iron acquisition in various environments, such as competition with other organisms on host surfaces, or sequestration by the high-affinity iron-binding host proteins lactoferrin and transferrin, growth of *V. anguillarum* is proficient in both host and marine habitats, achieved by multiple iron transport systems. Moreover, there is increasing evidence for the position of iron uptake, especially siderophores, at the crux of the microbial infection process, thereby reducing the appeal of siderophores as antimicrobial targets. In this context, it is important to investigate the whole of the iron acquisition systems of *V. anguillarum*, which will not only provide new insights to explain the evolutionary origin of versatile iron transport systems but also supply more evidence to understand the pathogenicity of this organism.

However, despite the exciting developments in understanding siderophore synthesis and ferrisiderophore transport in some vibrios, such as *V. anguillarum* and *V. cholerae*, there are many unsolved aspects of the iron uptake systems. For example, the mechanisms that govern the synthesis of anguibactin or vanchrobactin are still not fully elucidated, and how proteins are regulated for anguibactin or vanchrobactin synthesis is not yet well understood. How is mature siderophore secreted to the environment, and how is the iron released to the periplasm or cytoplasm from the ferric-siderophore complex? To answer these questions, an effective way is required to monitor anguibactin or vanchrobactin in real time. Finally, the different iron uptake systems that are responsible for the utilization of diverse iron sources will need to be established at the genetic level in order to uncover the relationship between iron uptake and virulence.

## Author contributions

All authors listed have made a substantial, direct and intellectual contribution to the work, and approved it for publication.

### Conflict of interest statement

The authors declare that the research was conducted in the absence of any commercial or financial relationships that could be construed as a potential conflict of interest.
