# Supplementary material for: Iron Acquisition Strategies of Vibrio anguillarum
Source: Front Cell Infect Microbiol. 2017 Jul 25;7:342. doi: 10.3389/fcimb.2017.00342 (PMC5524678; doi:10.3389/fcimb.2017.00342)
Supplement: Supplementary file 1 [file Table1.PDF]

Supplementary Material

The iron acquisition strategies in *Vibrio anguillarum*

Yingjie Li[1, 2], and Qingjun Ma[1, 2*]

[1]*Key Laboratory of Experimental Marine Biology, Institute of Oceanology, Chinese Academy of Sciences, Qingdao, China*
[2]*Laboratory for Marine Biology and Biotechnology, Qingdao National Laboratory for Marine Science and Technology, Qingdao, China*

***Correspondence to Qingjun Ma: qma@qdio.ac.cn**

Table S1 Summary of proteins known involved in iron utilization in *V. anguillarum*

Protein	Function	Roles in iron utilization	Reference
Anguibactin and vanchrobactin synthesis			
AngB/G	Isochorismate lyase and anguibactin assembly, NRPS family protein	Not essential for anguibactin synthesis	(Welch et al., 2000)
AngC	Isochorismate synthase	Not essential for anguibactin synthesis	(Alice et al., 2005)
AngD	Phosphopantheteinyl transferase	Essential for anguibactin synthesis	(Naka et al., 2008)
AngE	2,3-dihydroxybenzoate-AMP ligase; [a]NRPS family protein	Not essential for anguibactin synthesis	(Alice et al., 2005)
AngH	Histamine biosynthesis protein	Essential for anguibactin synthesis	(Barancin et al., 1998)
AngM	NRPS family protein	Essential for anguibactin synthesis	(Di Lorenzo et al., 2004)
AngN	NRPS family protein	Essential for anguibactin synthesis	(Di Lorenzo et al., 2008)
AngR	Anguibactin biosynthesis and regulation of iron uptake, NRPS family protein; Anguibactin synthesis regulator	Essential for anguibactin synthesis	(Salinas et al., 1989;Singer et al., 1991)
AngT	Thioesterase	Possibly involved in anguibactin degradation	(Wertheimer et al., 1999)
AngU	Putative monooxygenase	Possibly involved in N-hydro-histamine	(Naka et al., 2013a)
VabA	2,3-dihydro-2,3-dihydroxybenzoate-AMP ligase	Essential for anguibactin or vanchrobactin synthesis	(Alice et al., 2005;Balado et al., 2006)
VabB	Isochorismate lyase; NRPS family protein	Essential for vanchrobactin but not for anguibactin synthesis	(Balado et al., 2006)
VabC	Isochorismate synthase	Essential for vanchrobactin but not for anguibactin synthesis	(Alice et al., 2005;Balado et al., 2006)
VabD	Phosphopantheteinyl transferase	Essential for vanchrobactin but not for anguibactin synthesis	(Alice et al., 2005;Balado et

			al., 2006)
VabE	2,3-dihydroxybenzoate-AMP ligase; NRPS family protein	Essential for vanchrobactin but not for anguibactin synthesis	(Alice et al., 2005;Balado et al., 2006)
VabF	NRPS family protein	Essential for vanchrobactin synthesis	(Balado et al., 2006)
VabG	[b]DAHP synthetase	Not essential for vanchrobactin synthesis	(Balado et al., 2008)
VabH	Siderophore esterase	Possibly involved in vanchrobactin degradation	(Balado et al., 2006)
VabS	Putative vanchrobactin exporter	Possibly involved in the export of vanchrobactin	(Balado et al., 2006)
AroC	Chorismate synthase	Be deficient to produce 2,3-DHBA	(Chen et al., 1994)
Siderophore and heme import			
FatA	Outer membrane anguibactin receptor	Essential for anguibactin transport	(Walter et al., 1983;Singer et al., 1991)
FatB	Periplasmic binding lipoprotein	Essential for anguibactin transport	(Actis et al., 1995)
FatC	Iron-compound ABC transporter permease	Essential for anguibactin transport	(Naka et al., 2010)
FatD	Iron-compound ABC transporter permease	Essential for anguibactin transport	(Naka et al., 2010)
FatE	ATPase	Essential for anguibactin transport	(Naka et al., 2013b)
FvtA	Outer membrane siderophore receptor	Essential for vanchrobactin transport	(Balado et al., 2009;Naka and Crosa, 2012)
FvtB	Periplasmic binding protein	Essential for vanchrobactin transport	(Naka et al., 2013b)
FvtC	Iron-compound ABC transporter permease	Essential for vanchrobactin transport	(Naka et al., 2013b)
FvtD	Iron-compound ABC transporter permease	Essential for vanchrobactin transport	(Naka et al., 2013b)
FvtE	ATPase	Not essential for vanchrobactin transport	(Naka et al., 2013b)
FetA	Outer membrane enterobactin receptor	Not essential for enterobactin transport	(Naka and Crosa, 2012)
HuvA	Outer membrane heme receptor	Essential for heme uptake, but not essential for heme binding	(Mazoy et al., 2003)
HuvB	Periplasmic heme binding protein	Essential for heme uptake	(Mouriño et al., 2004)
HuvC	Inner membrane permease	Essential for heme uptake	(Mouriño et al., 2004)

HuvD	ATPase	Essential for heme uptake	(Mouriño et al., 2004)
HuvS	Outer membrane heme receptor	Heme uptake	(Mouriño et al., 2005)
HuvX	Putative heme delivery protein	Not essential for heme uptake	(Mouriño et al., 2004)
HuvZ	Putative heme degradation protein	Essential for heme uptake	(Mouriño et al., 2004)
TonB system			
TonB1	Energy transducting protein	Energy supply for anguibactin, vanchrobactin, or heme transport	(Stork et al., 2004)
ExbD1	Accessory protein	Energy supply for anguibactin, vanchrobactin, or heme transport	(Stork et al., 2004)
ExbB1	Accessory protein	Energy supply for anguibactin, vanchrobactin, or heme transport	(Stork et al., 2004)
TtpC	TonB2 complex-associated transport protein C	Essential for anguibactin or vanchrobactin transport	(Stork et al., 2007b)
ExbB2	Accessory protein	Essential for anguibactin or vanchrobactin transport	(Stork et al., 2004)
ExbD2	Accessory protein	Essential for anguibactin or vanchrobactin transport	(Stork et al., 2004)
TonB2	Energy transducting protein	Essential for anguibactin or vanchrobactin transport	(Stork et al., 2004)
Iron regulator			
TAFr	*trans*-acting factor	Positive regulator for anguibactin synthesis and transport	(Tolmasky et al., 1988)
RNAα	Antisense RNA	Negative regulator of anguibactin synthesis genes	(Waldbeser et al., 1993;Stork et al., 2007a)
Fur	Iron regulator	Global regulation of iron uptake	(Tolmasky et al., 1994)
VabR	LysR family regulator	Not essential for vanchrobactin synthesis	(Balado et al., 2008)
FetR	LysR family regulator	Regulator of *fetA* expression	(Naka and Crosa, 2012)

[a]NRPS, Nonribosomal peptide synthetase family protein

[b]DAHP, 3-deoxy-D-arabino-heptulosonate-7-phosphate

Table S2 BlastP analyses of proteins involved in siderophore secretion in *V. anguillarum* 775 using *P. aeruginosa or E. coli* as a query.

Protein	Function	GeneBank No. in the genome of *V. anguillarum* 775 (E-value, similarity)	Reference
PvdT	Pyoverdine export	AEH35172.1 (9e-77, 69%)	(Hannauer et al., 2010;Yeterian et al., 2010;Hannauer et al., 2012).
PvdR	Pyoverdine export	AEH34521.1 (1e-08, 41%)	
[a]MSF	Enterobactin export	AEH33648 (2e-132, 70%)	(Furrer et al., 2002)
[b]RND	Multidrug resistance; Enterobactin export	AEH31884 (1e-162, 52%)	(Furrer et al., 2002)
		AEH33753 (2e-139, 50%)	
		AEH34038 (5e-58, 45%)	
		AEH34520 (6e-49, 43%)	
		AEH35089 (1e-55, 43%)	
TolC	Channel protein	AEH32346 (9e-86, 62%)	(Furrer et al., 2002)
		AEH32997 (4e-06, 42%)	

[a]MSF: major facilitator superfamily protein;

[b]RND: resistance-nodulation-cell division.

Table S3 BlastP analyses of Feo, Fbp, and Fhu systems in vibrios and non-vibrio using *V. anguillarum* 775 as a query.

Protein in *V. anguillarum* 775	GenBank accession no.	Protein in different vibrios (e-value, similarity)					Best hit in non-*vibrio* (e-value, similarity)
		V. ordalii	*V. vulnificus*	*V. cholerae*	*V. alginolyticus*	*V. parahaemolyticus*	
FeoC	VAA_03398	WP_013856358.1 (2e-50, 100%)	WP_011149800.1 (2e-68, 62%)	WP_000602539.1 (1e-21, 53%)	WP_053311809.1 (1e-37, 82%)	WP_025554392.1 (4e-27, 62%)	*Vibrionales bacterium* SWAT-3 (4e-24, 55%)
FeoB	VAA_03399	WP_017048518.1 (0.0, 99%)	WP_072600083.1 (0.0, 80%)	WP_000877235.1 (0.0, 80%)	WP_053311810.1 (0.0, 88%)	WP_062865991.1 (0.0, 79%)	*Vibrionales bacterium* SWAT-3 (0.0, 77%)
FeoA	VAA_03400	WP_010318612.1 (4e-43, 97%)	WP_044127236.1 (2e-28, 69%)	WP_011149798.1 (4e-29, 71%)	WP_053311811.1 (5e-38, 83%)	WP_021822752.1 (6e-26, 68%)	*Vibrionales bacterium* SWAT-3 (2e-28, 72%)
FbpC-1	VAA_01820	WP_010318638.1 (5e-87, 46%)	WP_072617171.1 (3e-86, 52%)	WP_000414883.1 (0.0, 79%)	WP_046873805.1 (1e-90, 42%)	WP_029849463.1 (3e-88, 45%)	*Photobacterium gaetbulicola* (0.0, 83%)
FbpB-1	VAA_01822	WP_026027999.1 (0.0, 99%)	WP_045594837.1 (5e-48, 28%)	WP_069648649.1 (0.0, 81%)	WP_053304640.1 (5e-47, 28%)	WP_053808132.1 (9e-46, 28%)	*Photobacterium gaetbulicola* (0.0, 87%)
FbpA-1	VAA_01823	WP_069573401.1 (0.0, 99%)	WP_044024031.1 (2e-48, 36%)	WP_057563583.1 (0.0, 85%)	WP_031780463.1 (1e-48, 36%)	WP_069537516.1 (4e-49, 37%)	*Photobacterium marinum* (0.0, 88%)
FbpC-2	VAA_00770	WP_017049765.1 (0.0, 99%)	WP_080538702.1 (0, 81%)	WP_071179692.1 (0, 79%)	WP_053311297.1 (0, 85%)	WP_025500266.1 (0, 80%)	*Vibrionales bacterium* SWAT-3 (0.0, 80%)
FbpB-2	VAA_00769	WP_017045866.1 (0.0, 99%)	WP_045596826.1 (0.0, 80%)	WP_002043127.1 (0.0, 81%)	WP_053311298.1 (0.0, 86%)	WP_053809215.1 (0.0, 80%)	*Vibrionales bacterium* SWAT-3 (0.0, 78%)
FbpA-2	VAA_00768	WP_017045867.1 (0.0, 99%)	WP_017421219.1 （0.0, 80%）	WP_057555793.1 (0.0, 85%)	WP_053311299.1 (0.0, 93%)	WP_053809214.1 (0.0, 80%)	*Photobacterium halotolerans* (0., 80%)
FhuA	VAA_02423	WP_017050612.1	WP_046029546.1	WP_002030522.1	WP_047008473.1	WP_029825800.1	*Photobacterium profundum*

		(0.0, 99%)	(5e-71, 29%)	(0.0, 48%)	(0.0, 48%)	(0.0, 48%)	(0.0, 49%)
FhuC	VAA_02424	WP_026028190.1 (0.0，99%)	WP_060534593.1 （4e-82，49%）	WP_033929010.1 (4e-142，77%)	WP_017821854.1 (7e-134，70%)	WP_025508808.1 (1e-134，70%)	*Photobacterium profundum* （8e-134，72%）
FhuD	VAA_02425	WP_017050614.1 (0.0，99%)	WP_072610412.1 (1e-44，32%)	WP_000335753.1 (3e-137，63%)	WP_052708192.1 (3e-113，52%)	WP_062855498.1 (2e-114，58%)	*Photobacterium halotolerans* (2e-131，65%)
FhuB	VAA_02426	WP_017050615.1 (0.0，99%)	WP_045597356.1 (8e-114，37%)	WP_050902119.1 (0.0，73%)	WP_065645690.1 (0.0，60%)	WP_069536817.1 (0.0，61%)	*Desulfovibrio piezophilus* (0.0，66%)

References

Actis, L.A., Tolmasky, M.E., Crosa, L.M., and Crosa, J.H. (1995). Characterization and regulation of the expression of FatB, an iron transport protein encoded by the pJM1 virulence plasmid. *Mol. Microbiol.* 17**,** 197-204. doi: 10.1111/j.1365-2958.1995.mmi_17010197.x

Alice, A.F., Lopez, C.S., and Crosa, J.H. (2005). Plasmid- and chromosome-encoded redundant and specific functions are involved in biosynthesis of the siderophore anguibactin in *Vibrio anguillarum* 775: a case of chance and necessity? *J. Bacteriol.* 187**,** 2209-2214. doi: 10.1128/jb.187.6.2209-2214.2005

Balado, M., Osorio, C.R., and Lemos, M.L. (2006). A gene cluster involved in the biosynthesis of vanchrobactin, a chromosome-encoded siderophore produced by *Vibrio anguillarum*. *Microbiol-Sgm* 152**,** 3517-3528. doi: 10.1099/mic.0.29298-0

Balado, M., Osorio, C.R., and Lemos, M.L. (2008). Biosynthetic and regulatory elements involved in the production of the siderophore vanchrobactin in *Vibrio anguillarum*. *Microbiol-Sgm* 154**,** 1400-1413. doi: 10.1099/mic.0.2008/016618-0

Balado, M., Osorio, C.R., and Lemos, M.L. (2009). FvtA is the receptor for the siderophore vanchrobactin in *Vibrio anguillarum*: utility as a route of entry for vanchrobactin analogues. *Appl. Environ. Microbiol.* 75**,** 2775-2783. doi: 10.1128/AEM.02897-08

Barancin, C.E., Smoot, J.C., Findlay, R.H., and Actis, L.A. (1998). Plasmid-mediated histamine biosynthesis in the bacterial fish pathogen *Vibrio anguillarum*. *Plasmid* 39**,** 235-244. doi: 10.1006/plas.1998.1345

Chen, Q., Actis, L.A., Tolmasky, M.E., and Crosa, J.H. (1994). Chromosome-mediated 2,3-dihydroxybenzoic acid is a precursor in the biosynthesis of the plasmid-mediated siderophore anguibactin in *Vibrio anguillarum*. *J. Bacteriol.* 176**,** 4226-4234. doi:

Di Lorenzo, M., Poppelaars, S., Stork, M., Nagasawa, M., Tolmasky, M.E., and Crosa, J.H. (2004). A nonribosomal peptide synthetase with a novel domain organization is essential for siderophore biosynthesis in *Vibio anguillarum*. *J. Bacteriol.* 186, 7327-7336. doi: 10.1128/jb.186.21.7327-7336.2004

Di Lorenzo, M., Stork, M., Naka, H., Tolmasky, M.E., and Crosa, J.H. (2008). Tandem heterocyclization domains in a nonribosomal peptide synthetase essential for siderophore biosynthesis in *Vibrio anguillarum*. *Biometals* 21, 635-648. doi: 10.1007/s10534-008-9149-4

Furrer, J.L., Sanders, D.N., Hook-Barnard, I.G., and Mcintosh, M.A. (2002). Export of the siderophore enterobactin in *Escherichia coli*: involvement of a 43 kDa membrane exporter. *Mol. Microbiol.* 44, 1225-1234. doi: 10.1046/j.1365-2958.2002.02885.x

Hannauer, M., Braud, A., Hoegy, F., Ronot, P., Boos, A., and Schalk, I.J. (2012). The PvdRT-OpmQ efflux pump controls the metal selectivity of the iron uptake pathway mediated by the siderophore pyoverdine in *Pseudomonas aeruginosa*. *Environ Microbiol* 14, 1696-1708. doi: 10.1111/j.1462-2920.2011.02674.x

Hannauer, M., Yeterian, E., Martin, L.W., Lamont, I.L., and Schalk, I.J. (2010). An efflux pump is involved in secretion of newly synthesized siderophore by *Pseudomonas aeruginosa*. *FEBS Lett.* 584, 4751-4755. doi: 10.1016/j.febslet.2010.10.051

Mazoy, R., Osorio, C.R., Toranzo, A.E., and Lemos, M.L. (2003). Isolation of mutants of *Vibrio anguillarum* defective in haeme utilisation and cloning of *huvA*, a gene coding for an outer membrane protein involved in the use of haeme as iron source. *Arch. Microbiol.* 179, 329-338. doi: 10.1007/s00203-003-0529-4

Mouriño, S., Osorio, C.R., and Lemos, M.L. (2004). Characterization of heme uptake cluster genes in the fish pathogen *Vibrio anguillarum*. *J. Bacteriol.* 186, 6159-6167. doi: 10.1128/jb.186.18.6159-6167.2004

Mouriño, S., Rodriguez-Ares, I., Osorio, C.R., and Lemos, M.L. (2005). Genetic variability of the heme uptake system among different strains of the fish pathogen *Vibrio anguillarum*: identification of a new heme receptor. *Appl. Environ. Microbiol.* 71, 8434-8441. doi: 10.1128/Aem.71.12.8434-8441.2005

Naka, H., and Crosa, J.H. (2012). Identification and characterization of a novel outer membrane protein receptor FetA for ferric enterobactin transport in *Vibrio anguillarum* 775 (pJM1). *Biometals* 25, 125-133. doi: 10.1007/s10534-011-9488-4

Naka, H., Liu, M.Q., Actis, L.A., and Crosa, J.H. (2013a). Plasmid- and chromosome-encoded siderophore anguibactin systems found in marine vibrios: biosynthesis, transport and evolution. *Biometals* 26, 537-547. doi: 10.1007/s10534-013-9629-z

Naka, H., Liu, M.Q., and Crosa, J.H. (2013b). Two ABC transporter systems participate in siderophore transport in the marine pathogen *Vibrio anguillarum* 775 (pJM1). *FEMS Microbiol. Lett.* 341, 79-86. doi: 10.1111/1574-6968.12092

Naka, H., Lopez, C.S., and Crosa, J.H. (2008). Reactivation of the vanchrobactin siderophore system of *Vibrio anguillarum* by removal of a chromosomal insertion sequence originated in plasmid pJM1 encoding the anguibactin siderophore system. *Environ. Microbiol.* 10, 265-277. doi: 10.1111/j.1462-2920.2007.01450.x

Naka, H., Lopez, C.S., and Crosa, J.H. (2010). Role of the pJM1 plasmid-encoded transport proteins FatB, C and D in ferric anguibactin uptake in the fish pathogen *Vibrio anguillarum*. *Environmental microbiology reports* 2, 104-111. doi: 10.1111/j.1758-2229.2009.00110.x

Salinas, P.C., Tolmasky, M.E., and Crosa, J.H. (1989). Regulation of the iron uptake system in *Vibrio anguillarum*: evidence for a cooperative effect between two transcriptional activators. *Proc. Natl. Acad. Sci. U. S. A.* 86, 3529-3533. doi: DOI 10.1073/pnas.86.10.3529

Singer, J.T., Schmidt, K.A., and Reno, P.W. (1991). Polypeptides p40, pOM2, and pAngR are required for iron uptake and for virulence of the marine fish pathogen *Vibrio anguillarum* 775. *J. Bacteriol.* 173, 1347-1352.

Stork, M., Di Lorenzo, M., Mourino, S., Osorio, C.R., Lemos, M.L., and Crosa, J.H. (2004). Two tonB systems function in iron transport in *Vibrio anguillarum*, but only one is essential for virulence. *Infect. Immun.* 72, 7326-7329. doi: 10.1128/Iai.72.12.7326-7329.2004

Stork, M., Di Lorenzo, M., Welch, T.J., and Crosa, J.H. (2007a). Transcription termination within the iron transport-biosynthesis operon of *Vibrio anguillarum* requires an antisense RNA. *J. Bacteriol.* 189, 3479-3488. doi: 10.1128/jb.00619-06

Stork, M., Otto, B.R., and Crosa, J.H. (2007b). A novel protein, TtpC, is a required component of the TonB2 complex for specific iron transport in the pathogens *Vibrio anguillarum* and *Vibrio cholerae*. *J. Bacteriol.* 189, 1803-1815. doi: 10.1128/Jb.00451-05

Tolmasky, M.E., Actis, L.A., and Crosa, J.H. (1988). Genetic analysis of the iron uptake region of the *Vibrio anguillarum* plasmid pJM1: molecular cloning of genetic determinants encoding a novel trans activator of siderophore biosynthesis. *J. Bacteriol.* 170, 1913-1919.

Tolmasky, M.E., Wertheimer, A.M., Actis, L.A., and Crosa, J.H. (1994). Characterization of the *Vibrio anguillarum fur* gene: role in regulation of expression of the FatA outer membrane protein and catechols. *J. Bacteriol.* 176, 213-220.

Waldbeser, L.S., Tolmasky, M.E., Actis, L.A., and Crosa, J.H. (1993). Mechanisms for negative regulation by iron of the *fatA* outer membrane protein gene expression in *Vibrio anguillarum* 775. *J. Biol. Chem.* 268, 10433-10439.

Walter, M.A., Potter, S.A., and Crosa, J.H. (1983). Iron uptake system mediated by *Vibrio anguillarum* plasmid pJM1. *J. Bacteriol.* 156, 880-887.

Welch, T.J., Chai, S.H., and Crosa, J.H. (2000). The overlapping *angB* and *angG* genes are encoded within the *trans*-acting factor region of the virulence plasmid in *Vibrio anguillarum*: essential role in siderophore biosynthesis. *J. Bacteriol.* 182, 6762-6773. doi: 10.1128/jb.182.23.6762-6773.2000

Wertheimer, A.M., Verweij, W., Chen, Q., Crosa, L.M., Nagasawa, M., Tolmasky, M.E., et al. (1999). Characterization of the *angR* gene of *Vibrio anguillarum*: essential role in virulence. *Infect. Immun.* 67**,** 6496-6509.

Yeterian, E., Martin, L.W., Lamont, I.L., and Schalk, I.J. (2010). An efflux pump is required for siderophore recycling by *Pseudomonas aeruginosa*. *Environmental microbiology reports* 2**,** 412-418. doi: 10.1111/j.1758-2229.2009.00115.x
